# Callous–Unemotional Traits and Gun Violence: The Unique Role of Maternal Hostility

**DOI:** 10.3390/children12060775

**Published:** 2025-06-14

**Authors:** Nicholas D. Thomson, Sophie L. Kjærvik, Georgia Zacharaki, Abriana M. Gresham, Danielle M. Dick, Kostas A. Fanti

**Affiliations:** 1 Department of Surgery, Virginia Commonwealth University, Richmond, VA 23284, USA; abriana.gresham@vcuhealth.org; 2 Department of Psychology, Virginia Commonwealth University, Richmond, VA 23284, USA; 3 Norwegian Centre for Violence and Traumatic Stress Studies, 0484 Oslo, Norway; sophie.lyngesen.kjervik@nkvts.no; 4 Department of Psychology, University of Cyprus, Nicosia 2109, Cyprus; zacharaki.georgia@ucy.ac.cy (G.Z.); fanti.kostas@ucy.ac.cy (K.A.F.); 5 Department of Psychiatry, Rutgers University, Piscataway, NJ 08854, USA; danielle.m.dick@rutgers.edu

**Keywords:** callous-unemotional traits, parenting, violence, gun violence

## Abstract

**Background/Objectives:** Conduct Disorder (CD) involves persistent behavior violating societal norms and others’ rights. A subgroup of adolescents with CD exhibits callous–unemotional (CU) traits, which are linked to severe antisocial behavior and poorer long-term outcomes. Research suggests parenting plays an important role in the development of CU traits. However, few studies have explored the role of maternal and paternal parenting practices mediating the link between CU traits and violence within the same study. **Methods**: This study included 222 adolescents with Conduct Disorder (*M*_age_ = 15.7, 68% male) and examined whether perceived parental warmth and hostility, measured using the Quality of Parental Relationships, mediated the association between callous–unemotional (CU) traits and youth involvement in violent crime and gun violence, assessed via the Violent Crime Assessment and Gun Violence Questionnaire. **Results**: Double mediation models showed that only maternal hostility mediated the link between CU traits and violence, while maternal warmth and paternal parenting practices did not. **Conclusions**: Findings emphasize the role of maternal hostility in exacerbating violence, including gun violence, among adolescents with CD and CU traits.

## 1. Introduction

Conduct Disorder is one of the most prevalent childhood psychiatric disorders, with a global prevalence of ~3.2% [1,2]. Youth with CD are characterized by a chronic pattern of behavior that violates the rights of others outside of age-appropriate societal norms or rules [3]. Adolescents with CD may display aggressive and cruel behaviors toward people or animals and engage in property destruction, deceitfulness, theft, and a persistent violation of rules (i.e., running away from home [4]). Not only are adolescents with CD at greater risk of criminal offending, but they are also more likely to develop severe mental health problems [1,5] delinquency later in life, facing several social and occupational difficulties during adulthood [6,7]. CD differs from other externalizing disorders (e.g., Oppositional Defiant Disorder) because of its greater severity, risk of legal involvement, and stronger association with later antisocial personality disorder [8]. CD affects an estimated 9.5% of the U.S. population, with an onset typically occurring around 11 years old, with higher rates among males (12%) compared to females (7.1% [9,10]). This higher risk for CD among boys than girls is probably due to both biological (e.g., testosterone levels, neurodevelopmental vulnerabilities) and socialization factors (e.g., aggression is more accepted among boys [10]).

Among adolescents with CD, there is a subgroup (approximately ~20% [2]) who display callous–unemotional (CU) traits (also termed Limited Prosocial Emotions LPEs; [4]). CU traits are characterized by a callous lack of empathy, remorse, and guilt; shallow or deficient affect; and a lack of concern about performance [11,12]. While small in number, adolescents with CD and CU traits are at risk of more severe forms of aggressive and antisocial behavior and poorer mental health outcomes into adulthood [2,13,14]. Beyond age and gender, other sociodemographic factors may increase risk for CD, CU traits, and aggression. A lower socioeconomic status has been consistently linked to higher behavioral risks, possibly due to increased exposure to stress and community violence and limited access to mental health care [10,15]. Adolescence is a critical developmental period for understanding the persistence of CU traits and CD, as it marks a stage when personality traits become more crystallized and stable. During this time, adolescents encounter growing independence and diverse social challenges, yet their regulatory systems remain underdeveloped, making them particularly susceptible to environmental and interpersonal influences [16]. For instance, peer group affiliation plays a critical role in identity formation and social development during adolescence [17]. Adolescence with CD and CU traits may struggle to establish positive social relationships due to disruptive behaviors, which increases the risk of social rejection and alignment with deviant peer groups [18]. To improve treatment protocols for adolescents with CU traits, it is necessary to understand why they are at increased risk for antisocial outcomes (i.e., mediators). Understanding mediators that link CU traits to antisocial behaviors provides prevention scientists with specific targets to disrupt the mechanistic pathway associated with CU-related violence. One potential mechanism that can be targeted in interventions and warrants exploration is parenting practices.

### 1.1. Parenting Practices and CU Traits

Prior research indicates that early interventions focusing on parenting practices can minimize the risk for antisocial phenotypes [19]. However, the role of parenting practices in CU-related violence remains largely unexplored, and what does exist has mixed results [20,21]. Yet, most studies have shown that there is an association between experiencing negative parenting, CU traits, and antisocial behavior in adolescents [22], and it is well documented that environmental factors, particularly parenting, contribute to violent behavior [23]. Moreover, prior studies demonstrate that low parental warmth is related to CU traits. For example, Pasalich and colleagues examined the association between maternal and paternal warmth and CU traits in a sample of 95 children with conduct problems and found that higher levels of paternal warmth were associated with lower levels of CU traits [24], consistent with prior longitudinal research [25]. Additionally, a recent study with 754 participants found that secondary CU traits were related to low parental warmth [26]. Thus, parental warmth may be a significant predictor of child antisocial behavior, particularly at high levels of CU traits.

### 1.2. Theoretical Framework

Several theories support the role of parenting in either exacerbating or mitigating the link between CU traits and violence. According to social learning theory [27], children learn behaviors by observing their caregivers. When adolescents with CU traits are exposed to harsh or callous parenting, they may internalize these behaviors as acceptable responses to conflict and emotion [28,29]. In contrast, warm and responsive parenting offers alternative models for prosocial behavior, potentially reducing the risk of antisocial outcomes. Additionally, coercion theory [30] specifies that hostile parental responses can escalate child aggression, creating a coercive cycle that reinforces problem behavior. In youth with CU traits who are less sensitive to punishment [31], this cycle may intensify, as parents may increase punitive responses, further fueling violence. On the other hand, consistent warm parenting may interrupt this cycle by modeling alternative prosocial behaviors.

Similarly, attachment theory [32] proposes that secure attachment, an emotional bond formed through warm, sensitive, and consistent parenting, promotes emotional regulation, empathy, and prosocial behavior—key protective factors against violence. This early bond is critical for human development, as it shapes how children learn to manage distress, relate to others, and interpret social cues. Conversely, hostile, neglectful, or inconsistent parenting can lead to insecure attachment, heightening emotional dysregulation, impaired empathy, and the risk for externalizing problems, including aggression and violence [33]. Indeed, insecure attachment has been linked to CU traits [24,34,35]. Children with CU traits show deficits in attachment due to their diminished emotional responsiveness and limited sensitivity to relational cues. However, attachment theory also suggests that warm and responsive parenting may help foster more positive tendencies or at least reduce antisociality, even among children with CU traits. Based on these theories, we propose that it is important to account for both parental warmth and hostility when examining the pathways from CU traits to serious violence.

Indeed, a longitudinal study among young offenders has shown that parental warmth was associated with lower psychopathic traits, whereas parental hostility was associated with higher psychopathic traits [36]. Further, maternal warmth was linked to greater prosocial behavior and maternal hostility to increased aggression in adolescent males within the justice system [37]. However, there is no clear evidence in terms of how maternal warmth and hostility may mediate the link between CU traits and more severe forms of violence, such as violent crime and gun violence. This is important as parenting practices are malleable to treatment [38] and could break the link between CU traits and criminal violence. If this is the case, parenting programs targeting harsh parenting could be particularly effective in reducing CU-related violence.

The Parental Acceptance–Rejection Theory (PARTheory [39]) is also a compelling theory for understanding how parental behaviors such as warmth and hostility influence adolescents’ development of antisocial behavior. According to PARTheory, perceived parental acceptance, encompassing warmth and affection, promotes psychological well-being and prosocial development, while perceived rejection, including hostility and neglect, contributes to emotional dysregulation, impaired empathy, and increased externalizing behaviors. This theory is especially relevant to adolescents with CU traits, because these traits are thought to be shaped, in part, by early caregiver environments [18,40]. Thus, PARTtheory is a good framework for explaining how parental warmth and hostility may not only relate to the expression of CU traits but also function as potential mechanisms through which these traits contribute to violence.

Cascade models of developmental psychopathology suggest that early vulnerability, such as CU traits, can set off a chain of negative developmental events through their influence on the child’s environment [41]. These models emphasize that maladaptive outcomes emerge over time via dynamic, transactional processes. For example, CU traits may alter how children perceive or elicit parenting, leading to lower warmth or greater hostility, which in turn can increase the risk of violence. Thus, supporting the current study’s mediational model, where youth-perceived parenting is viewed as a key mechanism linking CU traits to violent behavior.

Although CU traits are well-established predictors of aggression and violence, the underlying mechanisms linking CU traits to violence remain less understood, and this is the first study to explore the role of parenting in CU-related gun violence. While prior research has examined parenting as a risk factor for CU traits or violence separately, few studies have tested parenting as a mediator of the CU–violence link in high-risk youth. The current study addresses this gap by testing a theoretically grounded, developmentally informed model in which perceived parenting practices mediate the link between CU traits and violent behavior. Clarifying this connection may help identify modifiable, family-based targets for violence prevention.

### 1.3. The Present Study

This study aimed to examine whether adolescents’ experience of parental warmth and hostility mediated the relationship between CU traits and violence and gun violence. The goal was to identify modifiable developmental mechanisms that could explain how CU traits lead to severe forms of aggression. Thus, parenting was conceptualized as a mediator rather than a moderator. Because our goal was to identify modifiable pathways linking CU traits to violence, we frame parenting as a developmental process through which CU traits lead to aggression, rather than as a contextual factor that alters the strength of this association. Theoretical models such as the social learning theory and coercion theory suggest that adolescents with CU traits may elicit more hostile parenting responses, which in turn may reinforce aggressive behavior patterns. This conceptualization shifts the focus from parenting as a background condition to a dynamic process that responds to and shapes adolescents’ behavior.

Our rationale for this direction of mediation—parenting as the pathway from CU traits to violence, rather than CU traits mediating the effects of parenting—is rooted in social learning theory [27]. This theory suggests that adolescents may model violent behavior when exposed to hostile parenting, particularly if they already possess traits that reduce empathy and remorse. On the other hand, warm parenting may provide a prosocial model and reinforce appropriate conduct, even in the presence of CU traits.

To advance prior work that has primarily focused on general aggressive behavior, we examine how parental warmth and hostility relate to violent crime and gun violence. While this is a relatively new line of inquiry, CU traits have been linked to higher levels of gun violence and risky gun behaviors [29,42,43]. Given that gun violence is one of the leading causes of death among adolescents [44], exploring mediators linking CU traits to gun violence is an important line of inquiry. We expect CU traits to be associated with violent crime and gun violence [29,45,46,47,48] and higher parental hostility and lower parental warmth to mediate these associations.

## 2. Materials and Methods

### 2.1. Participants

This study included 222 participants aged 12–17 (M_age_ = 15.68, *SD* = 1.36) with a diagnosis of CD. Most participants were male (68%) and identified as African American (52%), White (40%), American Indian or Alaska Native (1.4%), or mixed race (6.6%).

### 2.2. Procedure

Adolescent participants were recruited through a large healthcare network in Virginia by trained research coordinators and were required to have a CD diagnosis made by a licensed psychiatrist or psychologist. Before data collection commenced, adolescents and their caregivers provided assent and consent in separate and private rooms. This project is part of a larger study examining the stability of CU traits among adolescents with CD (R01MH123535: PI Thomson). Ethical approval for the study was granted by the Virginia Commonwealth University Institutional Review Board. Participants received USD 200 for their participation in the larger study. The assessments included in this study were collected during the baseline session. Participants were excluded from each model if they had incomplete responses or if they did not have the specified parental figure.

### 2.3. Measures

**Callous–Unemotional Traits:** The youth version of the Inventory of Callous–Unemotional Traits (ICU [49]) is a self-report scale developed to assess callous–unemotional traits in adolescence. The scale includes 24 items rated on a 3-point scale (1 = not at all true; 3 = definitely true). The scale includes positively and negatively worded items; negatively worded items were reverse-scored such that higher ratings indicate higher levels of CU traits. The ICU yielded good internal consistency in the present study (α = 0.81).

**Parental Hostility and Warmth:** The Quality of Parental Relationships Inventory (QPRI [50]) is a self-report measure developed to assess maternal and paternal hostility and warmth. The scale consists of 42 items (9 to assess maternal warmth (MW), 12 to assess maternal hostility (MH), 9 to assess paternal warmth (PW), and 12 to assess paternal hostility (PH)) that participants rate on a 4-point scale (1 = always; 4 = never). On the warmth subscale, higher scores indicate a more supportive and nurturing parental relationship. On the hostility subscale, higher scores indicate a more angry and hostile relationship. If adolescents did not have a maternal or paternal figure, cases were excluded. The scales showed good internal consistency (maternal hostility, α = 0.83; maternal warmth, α = 0.91; paternal hostility, α = 0.89; paternal warmth, α = 0.96).

**Violent Crime:** The Violent Crime Assessment is a self-report measure of violent crime perpetration and victimization. For the present study, only the perpetration scale was used. The scale includes 6 items that describe (1) simple assault (i.e., “How many times have you pushed, slapped, strangled or hit someone?”), (2) aggravated assault with a weapon (e.g., “How many times have you harmed someone with a dangerous weapon (e.g., knife, gun)?”), (3) homicide (e.g., “How many times have you killed another person?”), (4) robbery (e.g., “How many times have you taken something from another person, such as cash or their property by using force against them (whether or not they got hurt)?”), (5) rape (e.g., “How many times have you forced (physically or psychologically) another person to have sexual intercourse with you? This includes vaginal, anal or oral penetration.”), and (6) sexual assault (“How many times have you made sexual contact with a person who did not want the contact? Sexual contact includes grabbing or fondling.”). Each item is rated on a 6-point scale (1 = never, 2 = 1–2 times, 3 = 3–5 times, 4 = 6–9 times, 5 = 10–19 times, and 6 = 20 or more times). The VCA was worded to assess the lifetime occurrence of violent crime perpetration, and total scores on a full scale were used in analyses for the present study.

**Gun Violence:** The Gun Violence Questionnaire (GVQ) is a self-report measure with 15 items rated on a 3-point scale (0 = never; 2 = often). The scale measures two types of gun violence: reactive (6 items; e.g., “Used a gun when I was provoked by others”) and proactive gun violence (8 items; e.g., “Used a gun to take things from others”). Higher scores indicate more gun violence. The scale was developed based on the RPQ [51]. The scale had adequate internal consistency in this study (*a* = 0.77).

### 2.4. Data Analytics Plan

Zero-order correlations among the study variables were examined to understand the association between CU traits, violence types, and parental hostility and warmth. Double mediation analyses were conducted with CU traits as the predictor, parental hostility and warmth as mediators, and violent crime and gun violence as outcomes. Separate models were conducted for maternal and paternal relationships. Analyses were performed in PROCESS for R Version 4.3.1, enabling a regression-based mediation analysis approach with bootstrapping and robust standard errors. The bootstrapping mediation model was conducted with 10,000 replications. Age, sex (0 = female; 1 = male), and race (0 = other; 1 = African American) were included as covariates in all models. An a priori Monte Carlo Power Analysis for indirect effects [52] with a power set to 0.80 and medium effects showed a required sample size of 128. The GVQ was log-transformed to correct for non-normality, as the data exceeded acceptable thresholds for skewness (>3) and kurtosis (>10), with observed values of 4.43 and 23.58, respectively [53,54].

## 3. Results

### 3.1. Preliminary Analysis

The correlations and descriptive statistics are displayed in Table 1. CU traits were positively related to violent crime (r = 0.29, *p* < 0.001) and gun violence (r = 0.27, *p* < 0.001) and negatively related to maternal warmth (r = −0.19, *p* = 0.004). Violent crime was positively related to gun violence (r = 0.25, *p* < 0.001) and maternal hostility (r = 0.31, *p* < 0.001). Gun violence was positively related to maternal hostility (r = 0.17, *p* = 0.02). In addition, maternal hostility was negatively correlated with maternal warmth (r = −0.55, *p* < 0.001) and paternal warmth (r = −0.15, *p* = 0.04) and positively correlated with paternal hostility (r = 0.26, *p* < 0.001). Lastly, paternal warmth was positively correlated with maternal warmth (r = 0.34, *p* < 0.001) and negatively correlated with paternal hostility (r = −0.20, *p* = 0.004).

### 3.2. Maternal Parenting Practices

Double mediation models with maternal parenting mediators are displayed in Table 2.

**Figure 1:**
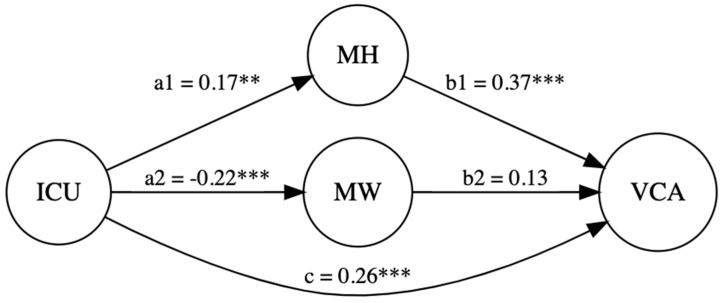
CU traits and violent crime with maternal hostility and warmth as mediators. *Note. p* < 0.01 **; *p* < 0.001 ***.

**Violent Crime:** CU traits (β = 0.17, *p* = 0.006) and sex (β = −0.39, *p* < 0.001) had a significant direct effect on maternal hostility: R^2^ = 0.18, F(4, 211) = 11.82, *p* < 0.001. Additionally, CU traits (β = −0.22, *p* < 0.001) and sex (β = 0.31, *p* < 0.001) had a significant direct effect on maternal warmth: R^2^ = 0.14, F(4, 211) = 8.71, *p* < 0.001. Further, CU traits (β = 0.26, *p* < 0.001) and maternal hostility (β = 0.37, *p* < 0.001) had a significant direct effect on violent crime: R^2^ = 0.18, F(6, 209) = 7.89, *p* < 0.001. CU traits also significantly indirectly affected violent crime via maternal hostility: β = 0.06, 95% CI [0.01, 0.14]. However, CU traits did not indirectly affect violent crime via maternal warmth (β = −0.03, 95% CI [−0.07, 0.002]). Thus, maternal hostility partially mediated the relation between CU traits and violent crime, and maternal warmth did not (see Figure 1).

**Figure 2:**
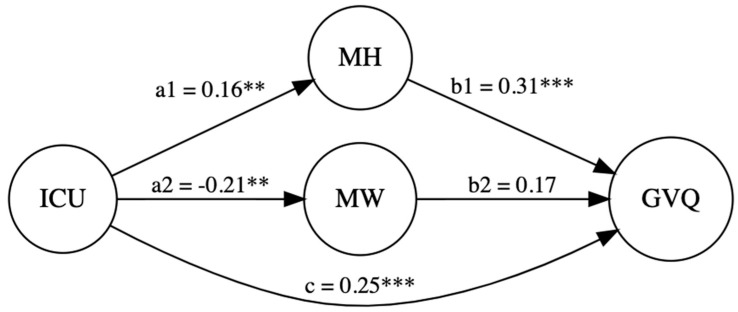
CU traits and gun violence with maternal hostility and warmth as mediators. *Note. p* < 0.01 **; *p* < 0.001 ***.

**Gun Violence:** CU traits (β = 0.16, *p* = 0.01) and sex (β = −0.40, *p* < 0.001) had a significant direct effect on maternal hostility: R^2^ = 0.19, F(4, 205) = 11.68, *p* < 0.001. CU traits (β = −0.21, *p* = 0.002) and sex (β = 0.32, *p* < 0.001) also had a significant direct effect on maternal warmth: R^2^ = 0.14, F(4, 205) = 8.52, *p* < 0.001. Moreover, CU traits (β = 0.25, *p* < 0.001), maternal hostility (β = 0.31, *p* < 0.001), and sex (β = 0.23, *p* = 0.002) had a significant direct effect on gun violence: R^2^ = 0.17, F(6, 203) = 7.02, *p* < 0.001. Lastly, CU traits had a significant indirect effect on gun violence via maternal hostility, β = 0.05, 95% CI [0.007, 0.10], but not via maternal warmth (β = −0.03, 95% CI [−0.08, 0.0005]). Thus, maternal hostility partially mediated the relation between CU traits and gun violence, while maternal warmth did not (see Figure 2).

### 3.3. Paternal Parenting Practices

The mediation models with paternal hostility and warmth are displayed in Table 3.

**Violent Crime:** CU traits did not directly predict paternal hostility (β = 0.10, *p* = 0.17), and the model was not significant: R^2^ = 0.03, F(4, 197) = 1.30, *p* = 0.27. In addition, CU traits (β = −0.10, *p* = 0.14) did not predict paternal warmth, but sex did (β = 0.23, *p* = 0.001), and the model was significant: R^2^ = 0.07, F(4, 197) = 3.60, *p* = 0.007. However, CU traits (β = 0.32, *p* < 0.001) did significantly and directly affect violent crime: R^2^ = 0.12, F(6, 195) = 4.30, *p* < 0.001. None of the mediators were significant. Thus, paternal hostility and warmth did not mediate the relation between CU traits and violent crime.

**Gun Violence:** CU traits did not directly predict paternal hostility (β = 0.08, *p* = 0.27), and the model was not significant: R^2^ = 0.02, F(4, 191) = 1.15, *p* = 0.33. In addition, CU traits (β = −0.10, *p* = 0.15) did not predict paternal warmth, but sex did (β = 0.23, *p* = 0.001), and the model was significant: R^2^ = 0.07, F(4, 191) = 3.69, *p* = 0.006. Further, CU traits (β = 0.27, *p* < 0.001) and sex (β = 0.17, *p* = 0.02) had a significant direct effect on gun violence: R^2^ = 0.12, F(6, 189) = 4.19, *p* < 0.001. However, none of the mediations were significant. Thus, paternal hostility and warmth did not mediate the relationship between CU traits and gun violence.

## 4. Discussion

This study extends prior research by demonstrating that adolescents with CD and CU traits are at greater risk of severe violence, including violent crime and gun violence. It adds to the literature on CU traits and parental practices by showing that maternal hostility, but not paternal hostility or parental warmth, mediated the association between CU traits and severe violence. The results align with research suggesting that harsh and hostile maternal parenting practices exacerbate antisocial behaviors in adolescents with CU traits [55,56]. However, the results also somewhat diverge from studies showing the protective effect of maternal warmth [37].

The findings lend support to PARTheory [39], which posits that perceived parental rejection (e.g., hostility) may be more psychologically impactful than the absence of warmth, particularly for emotionally unresponsive individuals [39]. For adolescents with CU traits who already struggle with empathy and closeness, maternal hostility may reinforce existing schemas of rejection, deepening detachment and dysregulation [31]. Coercion theory further suggests that these adolescents may be unresponsive to standard discipline, leading caregivers to escalate hostility in a pattern that reinforces antisocial behavior [30]. While the cross-sectional design limits causal conclusions, this interpretation shows the potential for coercive cycles in families with CD- and CU-affected adolescents.

The significance of maternal, but not paternal, hostility may reflect differences in caregiving roles. Mothers are often primary caregivers, particularly in high-risk families, and thus may serve as a more salient behavioral model [57]. Social learning theory supports this perspective, suggesting that adolescents are most likely to model the behavior of emotionally significant caregivers [28]. Thus, frequent exposure to maternal hostility may provide a template for conflict resolution rooted in aggression, at least if the maternal caregiver is more present or emotionally engaged. Developmental theory also emphasizes adolescence as a critical window for peer group affiliation, autonomy, and social learning [41]. During this stage, maternal hostility may reinforce an antisocial identity.

Importantly, these findings should not be interpreted as dismissing the role of paternal caregivers. The absence of significant paternal relations may reflect lower paternal behavior, lower day-to-day involvement, and less emotional proximity, or caregiver dynamics are not captured by warmth and hostility alone [58]. Additionally, adolescents in this study may have had limited paternal contact, which could impact both their behavior and their perceptions of parenting. Given that prior research has linked paternal absence, including through incarceration, to elevated psychopathic traits in adult offspring [59], future studies should investigate the distinct and potentially interactive roles of paternal caregivers, especially within diverse, high-risk family contexts where disrupted father–child relationships may contribute to maladaptive personality development.

The lack of findings for maternal and paternal warmth also deserves attention. Neither variable mediated the CU–violence association nor was it directly related to violence. This is consistent with some prior work showing no protective effect of paternal warmth on general delinquency [36] but contrasts with other findings showing that warmth mitigates antisocial outcomes [19,35]. One interpretation is that, for youth with CU traits, the presence of hostility may be more developmentally consequential than the absence of warmth. These youth may also perceive warmth as insincere or inconsistent, especially in families with ongoing conflict or dysregulation [60]. Additionally, the severity of CD in our samples may diminish the influence of warmth, even when present.

This is one of the first studies to explore parental warmth and hostility in relation to severe violence outcomes, including gun violence. While most prior research has emphasized risk factors, some recent evidence shows that family-level protective factors, such as mutual respect and emotional support, can buffer against gun violence in high-risk adolescents [61]. This suggests that broader family dynamics, beyond single dimensions of parenting, may play an important role in shaping violence trajectories. 

Given the mediating role of maternal hostility, interventions targeting negative parenting practices, particularly maternal hostility, may be key in preventing serious violence among adolescents with CU traits. Evidence-based parenting programs that promote positive discipline and strengthen the parent–child relationship [38] may be especially valuable. Additionally, addressing caregiver stress could indirectly reduce hostility. Chronic stress has been linked to harsher, more punitive parenting styles [62,63], and interventions that support stress reduction and burnout prevention may benefit from comprehensive family-level strategies that reduce parents’ ability to engage in positive parenting and therapeutic processes [64].

## 5. Limitations

This study has limitations that should be acknowledged. Due to the limited sample size, we could not test for sex differences in associations, which may have produced different results for maternal and paternal parenting practices. Although we used validated and widely used measures, these assessments were all self-reported, introducing the potential for common method variance. In addition, our data is cross-sectional, which restricts our ability to draw causal inferences. Although our findings are consistent with theoretical models proposing that parenting mediated the link between CU traits and violence, the directionality of these associations cannot be confirmed. Future research is needed to test if parenting has a long-term impact on CD- and CU-trait-related violence.

## 6. Conclusions

This study provides evidence that maternal hostility is a key factor linking CU traits to violent outcomes, including both violent crime and gun violence. Among adolescents with CD and CU traits, hostile maternal behaviors may play a particularly influential role in reinforcing patterns of violence. While parental warmth is often considered a protective factor, our results suggest that, in high-risk populations, the presence of hostility may be more influential than the absence of warmth. Although paternal parenting practices did not show significant effects in this study, these findings should be interpreted with caution. The lack of observed association does not necessarily imply that fathers are uninvolved or irrelevant; rather, it may reflect differences in caregiver roles, adolescents’ perceptions, or other contextual factors. Future research with more nuanced measures of father involvement is needed to clarify the unique role of fathers. These findings are consistent with theoretical frameworks such as social learning theory and coercion theory, which propose that children learn behavioral patterns through exposure to caregiver modeling and that negative parent–child interactions can escalate over time. For adolescents who already show emotional detachment and reduced sensitivity to punishment, maternal hostility may further encourage antisocial and violent behaviors.

From a prevention and intervention perspective, these results point to the importance of addressing maternal hostility in intervention programs for adolescents with CD and CU traits. Interventions that focus on reducing harsh parenting and improving parent–child interactions may be particularly effective in reducing the risk of severe violence. In addition, reducing caregiver stress may support more consistent and less punitive parenting, enhancing the effectiveness of family-based approaches. Overall, this study contributes to a growing understanding of how family dynamics influence the course of conduct problems and violence. By identifying maternal hostility as a modifiable target, this research supports the development of focused interventions aimed at reducing serious antisocial outcomes among high-risk adolescents.

## Figures and Tables

**Table 1:** Correlation and descriptive statistics for study variables.

	1	2	3	4	5	6	7	8	9	10
Age	-									
2. Sex ^a^	0.004	-								
3. Race ^a^	0.04	−0.18 *	-							
4. ICU	0.05	0.08	0.07	-						
5. VCA	−0.01	−0.04	0.11	0.29 **	-					
6. GVQ ^b^	−0.001	0.19 *	−0.11	0.27 **	0.25 **	-				
7. MH	0.13	−0.36 **	−0.007	0.12	0.31 **	0.17 *	-			
8. MW	−0.13	0.27 **	0.01	−0.19 *	−0.10	0.02	−0.55 **	-		
9. PH	0.09	−0.09	−0.04	0.08	0.003	0.06	0.26 **	−0.06	-	
10. PW	−0.09	0.20 *	−0.06	−0.08	−0.02	0.02	−0.15 *	0.34 **	−0.20 *	-
Mean	15.68	0.68	0.57	22.87	1.31	0.27	4.80	20.08	3.88	16.06
*SD*	1.36	0.47	0.50	8.49	1.72	0.54	3.60	6.17	4.50	8.88
Skewness	−0.58	−0.77	−0.29	0.70	1.55	2.24	1.77	−0.79	2.13	−0.39
Kurtosis	−0.86	−1.42	−1.92	0.61	2.05	4.74	4.09	−0.19	5.38	−1.11

*Note*. Spearman’s correlation ^a^; log-transformed ^b^. *p* < 0.05 *; *p* < 0.001 **.

**Table 2:** Maternal hostility and warmth as mediators between CU traits and violence.

Pathways	Estimate	*SE*	95% CI	
			Lower	Upper
**Violent Crime**				
*Total effect*	0.06 *	0.01	0.03	0.09
*Direct effects*				
CU traits → maternal hostility	0.07 *	0.03	0.02	0.13
CU traits → maternal warmth	−0.09 *	0.05	−0.26	−0.07
CU traits → violent crime	0.05 *	0.01	0.03	0.08
Maternal hostility → violent crime	0.18 *	0.04	0.10	0.26
Maternal warmth → violent crime	0.04	0.02	−0.003	0.08
*Indirect effect*				
CU traits → maternal hostility → violent crime	0.01 *	0.007	0.01	0.14
CU traits → maternal warmth → violent crime	−0.006	0.004	−0.07	0.002
**Gun Violence**				
*Total effect*	0.02 *	0.004	0.008	0.02
*Direct effects*				
CU traits → maternal hostility	0.07 *	0.03	0.02	0.12
CU traits → maternal warmth	−0.15 *	0.05	−0.25	−0.07
CU traits → gun violence	0.02 *	0.004	0.007	0.02
Maternal hostility → gun violence	0.05 *	0.02	0.02	0.08
Maternal warmth → gun violence	0.01	0.008	−0.0002	0.03
*Indirect effect*				
CU traits → maternal hostility → gun violence	0.003 *	0.002	0.007	0.007
CU traits → maternal warmth → gun violence	−0.002	0.001	−0.006	0.000

*Note*. *p* < 0.05 *; Sex, age, and race were adjusted as covariates. *SE* = bootstrap standard error; 95% CI = 95% bootstrap confidence intervals. Number of excluded cases due to missing data: violent crime = 6; gun violence = 12.

**Table 3:** Paternal hostility and warmth as mediators between CU traits and violence.

Pathways	Estimate	*SE*	95% CI	
			Lower	Upper
**Violent Crime**				
*Total effect*	0.06 *	0.01	0.04	0.09
*Direct effects*				
CU traits → paternal hostility	0.05	0.04	−0.02	0.13
CU traits → paternal warmth	−0.11	0.08	−0.28	0.05
CU traits → violent crime	0.06 *	0.01	0.04	0.09
Paternal hostility →violent crime	−0.008	0.02	−0.05	0.04
Paternal warmth → violent crime	0.006	0.23	−0.02	0.04
*Indirect effect*				
CU traits → paternal hostility → violent crime	−0.0004	0.001	−0.004	0.002
CU traits → paternal warmth → violent crime	−0.0007	0.002	−0.006	0.004
**Gun Violence**				
*Total effect*	0.02 *	0.004	0.01	0.03
*Direct effects*				
CU traits → paternal hostility	0.04	0.04	−0.03	0.12
CU traits → paternal warmth	−0.10	0.08	−0.27	0.05
CU traits → gun violence	0.02 *	0.005	0.009	0.03
Paternal hostility → gun violence	0.007	0.007	−0.02	0.03
Paternal warmth → gun violence	0.001	0.0006	−0.01	0.01
*Indirect effect*				
CU traits → paternal hostility → gun violence	0.0003	0.0008	−0.0009	0.003
CU traits → paternal warmth → gun violence	−0.0001	0.0008	−0.002	0.002

*Note. p* < 0.05 *; Sex, age, and race were adjusted as covariates. *SE* = bootstrap standard error; 95% CI = 95% bootstrap confidence intervals. Number of excluded cases due to missing data: violent crime = 24; gun violence = 30.

## Data Availability

The data supporting the findings of this study are not publicly available due to the sensitive nature and vulnerability of the sample population. However, the data may be made available from the corresponding author upon reasonable request.
